# Development of a system to analyze oral frailty associated with Alzheimer's disease using a mouse model

**DOI:** 10.3389/fnagi.2022.935033

**Published:** 2022-08-02

**Authors:** Eriko Kuramoto, Ayano Kitawaki, Takakazu Yagi, Hiroshi Kono, Shin-Ei Matsumoto, Hiromitsu Hara, Yasumasa Ohyagi, Haruki Iwai, Atsushi Yamanaka, Tetsuya Goto

**Affiliations:** ^1^Department of Oral Anatomy and Cell Biology, Graduate School of Medical and Dental Sciences, Kagoshima University, Kagoshima, Japan; ^2^Department of Oral Health Science, Kobe Tokiwa University, Kobe, Japan; ^3^Department of Biomaterials Science, Graduate School of Medical and Dental Sciences, Kagoshima University, Kagoshima, Japan; ^4^Department of Immunology, Graduate School of Medical and Dental Sciences, Kagoshima University, Kagoshima, Japan; ^5^Department of Neurology and Geriatric Medicine, Ehime University Graduate School of Medicine, Ehime, Japan

**Keywords:** oral frailty, Alzheimer's disease, triple transgenic mouse model of Alzheimer's disease, pathology, mesencephalic trigeminal nucleus, mastication, bite force, electromyography

## Abstract

The rapid aging of the population makes the detection and prevention of frailty increasingly important. Oral frailty has been proposed as a novel frailty phenotype and is defined as a decrease in oral function coexisting with a decline in cognitive and physical functions. Oral frailty has received particular attention in relation to Alzheimer's disease (AD). However, the pathomechanisms of oral frailty related to AD remain unknown. It is assumed that the mesencephalic trigeminal nucleus (Vmes), which controls mastication, is affected by AD pathology, and as a result, masticatory function may be impaired. To investigate this possibility, we included male 3 × Tg-AD mice and their non-transgenic counterpart (NonTg) of 3–4 months of age in the present study. Immunohistochemistry revealed amyloid-β deposition and excessive tau phosphorylation in the Vmes of 3 × Tg-AD mice. Furthermore, vesicular glutamate transporter 1-immunopositive axon varicosities, which are derived from Vmes neurons, were significantly reduced in the trigeminal motor nucleus of 3 × Tg-AD mice. To investigate whether the AD pathology observed in the Vmes affects masticatory function, we analyzed electromyography of the masseter muscle during feeding. The 3 × Tg-AD mice showed a significant delay in masticatory rhythm compared to NonTg mice. Furthermore, we developed a system to simultaneously record bite force and electromyography of masseter, and devised a new method to estimate bite force during food chewing in mice. Since the muscle activity of the masseter showed a high correlation with bite force, it could be accurately estimated from the muscle activity. The estimated bite force of 3 × Tg-AD mice eating sunflower seeds was predominantly smaller than that of NonTg mice. However, there was no difference in masseter weight or muscle fiber cross-sectional area between the two groups, suggesting that the decreased bite force and delayed mastication rhythm observed in 3 × Tg-AD mice were not due to abnormality of the masseter. In conclusion, the decreased masticatory function observed in 3 × Tg-AD mice was most likely caused by AD pathology in the Vmes. Thus, novel quantitative analyses of masticatory function using the mouse model of AD enabled a comprehensive understanding of oral frailty pathogenesis.

## Introduction

The population is aging rapidly worldwide, and it is estimated that by 2050, there will be 2 billion people aged 65 years and older (Kinsella and Phillips, 2005), and this large number of elderly people poses serious issues for the planning and provision of health and social care. Detection and prevention of frailty are key to solving this issue. Frailty is a state in which the vitality of the body and mind (e.g., motor and cognitive functions) declines with age and is affected by the coexistence of multiple chronic diseases, resulting in impaired daily functioning and the emergence of physical and mental fragility, while maintaining and improving daily functioning through appropriate intervention and support (Fried et al., 2001; Bauer and Sieber, 2008; Clegg et al., 2013; Hoogendijk et al., 2019). Between 25 and 50% of people older than 85 years are estimated to experience frailty, and these people have substantial risks of falls, disability, long-term care, and death (Fried et al., 2001; Song et al., 2010).

Oral frailty has been recently proposed as a novel frailty phenotype and defined as a decrease in oral function coexisting with a decline in cognitive and physical functions (Watanabe et al., 2017; Tanaka et al., 2018; Dibello et al., 2021a). Oral frailty is currently garnering increasing attention, particularly in relation to Alzheimer's disease (AD) (Dibello et al., 2021a; Nakamura et al., 2021b). Recent clinical studies have shown that impaired masticatory function is associated with mild cognitive impairment and AD (Miura et al., 2003; Gatz et al., 2006; Okamoto et al., 2010; Stein et al., 2010; Mummolo et al., 2014); moreover, masticatory function declines in proportion to cognitive decline (Campos et al., 2017; Ikebe et al., 2018; Kim et al., 2020). However, the causal relationship and pathomechanisms between cognitive decline and impaired masticatory function remain unclear.

Recently, AD pathology has been observed not only in the cortex but also in the brainstem (Parvizi et al., 2001; Uematsu et al., 2018), and it has been reported that the volume of the brainstem of AD patients is significantly smaller than that of normal subjects (Lee et al., 2015). Furthermore, changes in the brainstem have been observed since the early stages of dementia (Dutt et al., 2020, 2021). Because the brainstem is important for the control of jaw movements (Lund, 1991), it is possible that the brainstem is impaired by AD pathology, resulting in a decrease in masticatory function. To examine this hypothesis, it is essential to conduct a histopathological analysis of the brainstem and evaluation of masticatory function in parallel. However, it is impossible to perform highly invasive histopathological analysis in humans. Accordingly, there is a need to elucidate the pathological mechanism of impaired masticatory function using mouse models of dementia.

Various methods have been proposed to evaluate oral frailty in humans by examining the condition of the teeth and mouth, and oral functions. The evaluation items of tooth and mouth condition include the number of natural and functional teeth, tongue thickness as an index of oral nutritional status, and turbidity of mouthwash water as an index of oral hygiene status. Assessment items for oral function include maximum occlusal force, masticatory capacity (an indicator of general masticatory ability), maximum tongue pressure, repeated saliva drinking test, tongue-lip motor function test (oral diadochokinesis) with three sounds (pa, ta, and ka), and oral wetting level (Tanaka et al., 2018). In mice, the oral function is particularly difficult to assess, and only a few studies have measured maximum bite force (Okuda-Akabane et al., 1997; Kim et al., 2021). It remains unclear whether the maximum bite force of mice is an adequate assessment of oral function, because the maximum bite force is strongly influenced by the emotion and personality of mice (Kuchiiwa and Kuchiiwa, 2014).

A triple transgenic mouse model of AD (3 × Tg-AD) mice harbors human APP_Swe_, PS1_M146V_, and Tau_P301L_ gene mutations and has been reported to express AD pathology in the brainstem, preceding the cerebral cortex and hippocampus (Oddo et al., 2003a,b; Overk et al., 2009). In the present study, we investigated whether AD pathology in the brainstem of 3 × Tg-AD mice affects the masticatory function. We evaluated the intrinsic oral function of 3 × Tg-AD mice using a novel method, and performed histological analysis of the masseter muscles and immunohistochemical analysis of AD pathology by focusing on trigeminal nuclei, which are important for oral sensation (Waite, 2004) and mastication (Travers, 2004). A comprehensive evaluation of these results revealed that 3 × Tg-AD mice develop an impaired masticatory function that may be due to AD pathology in the trigeminal nuclei.

## Materials and methods

### Animals

All procedures involving animals were performed in accordance with the National Institutes of Health Guide for the Care and Use of Laboratory Animals. Mice were maintained under a 12-h light/dark cycle and provided access to both food and water *ad libitum*. The experiments were approved by the Committees for Animal Care (D19005) and the Recombinant DNA Study (S29017) at Kagoshima University (Kagoshima, Japan). A total of 41 homozygous 3 × Tg-AD (*n* = 21) and non-transgenic (NonTg; C57BL/6J, Japan CLEA Co., Ltd., Tokyo, Japan; *n* = 20) male mice of 2–4 months of age in a C57BL/6J background (the transfer is accomplished by at least 10 cycles of backcrossing) were used in the present study. Since the hormone levels of female mice fluctuate with the sexual cycle, which affects AD pathology and muscles (Carroll et al., 2007), we used male mice, which are presumed to exhibit less hormonal fluctuation. All efforts were made to minimize animal suffering and reduce the number of animals used in the present study.

### Surgical implantation

Mice were prepared for long-term recording of masseter muscles using electromyography (EMG). Surgery was performed under anesthesia using medetomidine (0.15 mg/kg; Kobayashi Kako, Fukui, Japan), midazolam (2.0 mg/kg; Astellas Pharma, Tokyo, Japan), and butorphanol (2.5 mg/kg; Meiji Seika Pharma Co., Ltd., Tokyo, Japan) (Kurihara et al., 2013). Two urethane-coated silver wires (diameter: 0.2 mm, Unique Medical, Tokyo, Japan) were implanted into the right masseter muscle, spaced 3 mm apart, and one wire was inserted into the back as a reference. The three wires for EMG were soldered to a three-pin pin-header for use as connectors (XJ8B0311; OMRON Co., Kyoto, Japan). The connector and wires were firmly fixed to the skull using dental cement (Super-Bond C&B; Sun Medical Co., Shiga, Japan) and dental acrylic resin (Natural Ortho Dontic Resin; Nissin Dental Products Inc., Kyoto, Japan).

### Bite force and EMG recording

**Figure 1:**
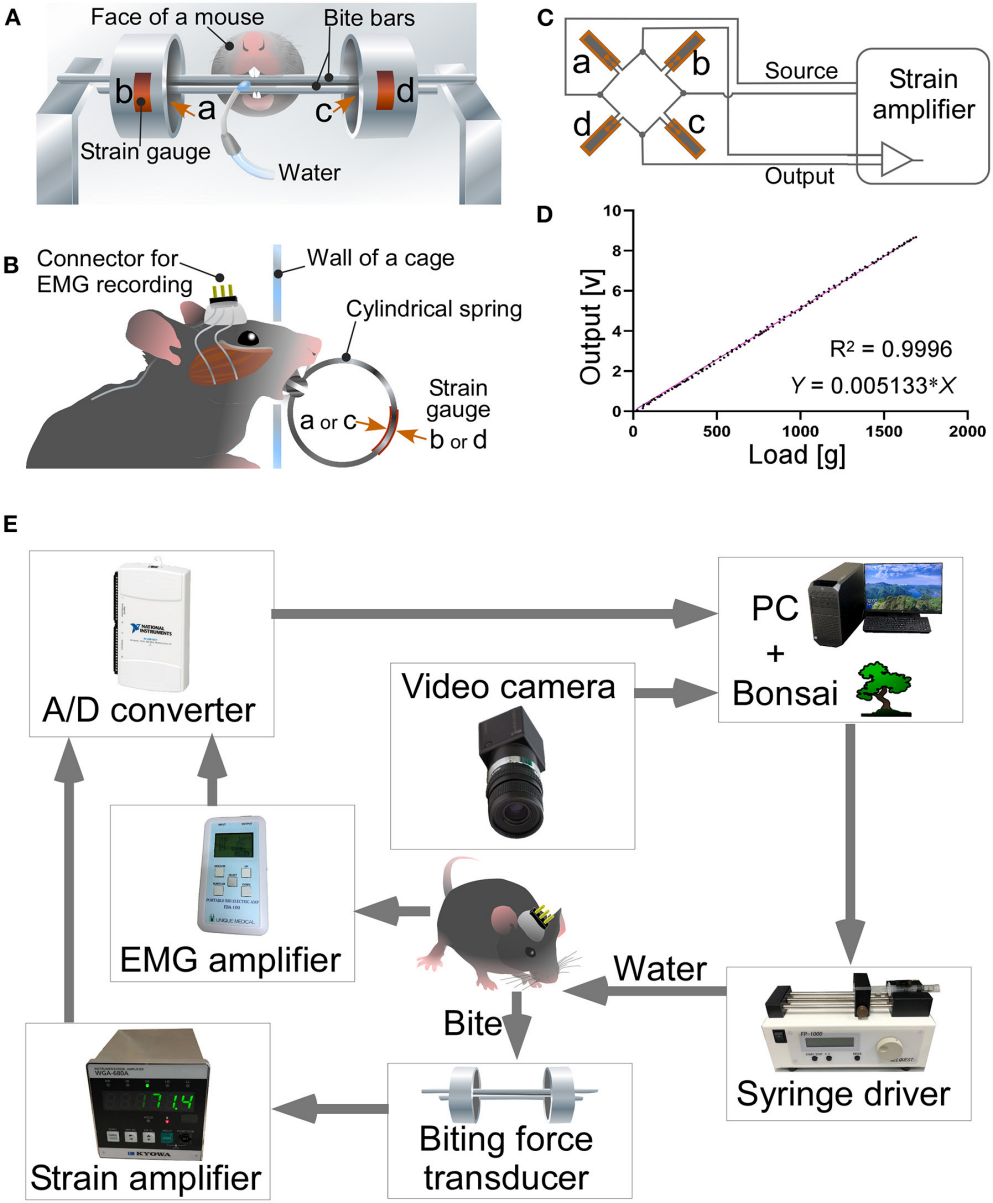
A bite force measurement apparatus and a system for simultaneous measurement of bite force and EMG. Front **(A)** and side views **(B)** of a bite force transducer. Strain gauges (b,d) were pasted on the outer surface of each cylindrical spring, while others (a,c) were placed on the inner surface. The wall of the recording cage has a hole of 1.5 cm in diameter. Mice faced through the hole, chewed bite bars, and drank the rewarding water. **(C)** A full bridge was constructed with four strain gages and connected to the strain amplifier. **(D)** Relationship between load and voltage output of the bite force transducer. **(E)** A system for simultaneous measurement of bite force and EMG. See text for further details.

**Figure 2:**
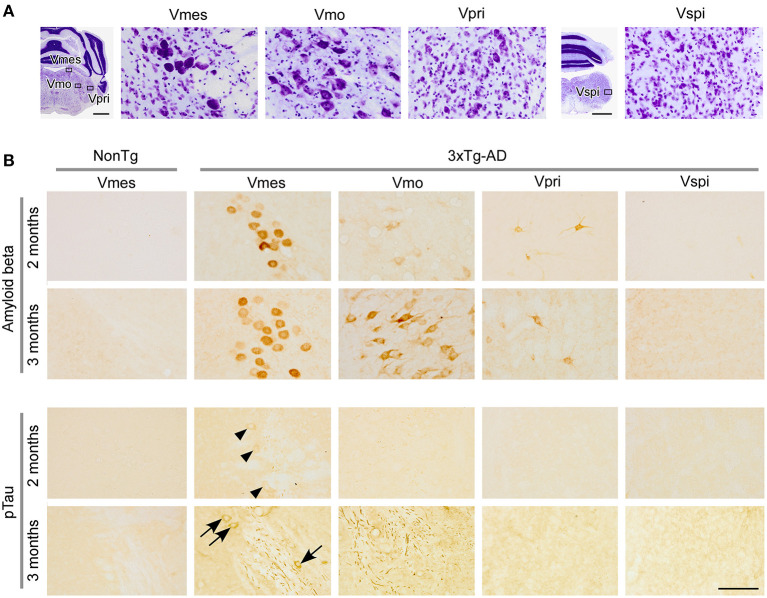
AD pathology in the trigeminal nuclei of 2- and 3-month-old 3 × Tg-AD mice. **(A)** Cytoarchitectonic structures of the trigeminal nuclei; the mesencephalic trigeminal nucleus (Vmes), trigeminal motor nucleus (Vmo), principal sensory trigeminal nucleus (Vpri), and spinal trigeminal nucleus (Vspi). **(B)** Intense Aβ immunoreactivity was observed in the Vmes and Vmo of 3 × Tg-AD mice. Weak Aβ immunoreactivity was also observed in the Vpri. In the Vmes of 2-month-old 3 × Tg-AD mice, p-tau weakly positive cell bodies (arrowheads) were observed; in 3-month-old 3 × Tg-AD mice, intense immunoreactivity to p-tau was observed in cell bodies (arrows) and axon fibers. In the Vmo, many p-tau-positive axon fibers (potentially derived from the Vmes) were observed. In NonTg mice, neither Aβ (human) nor p-tau immunoreactivity was observed. Scale bars: 1 mm **(A)** and 100 μm (in **B**; applies to A_Vmes_, A_Vmo_, A_Vpri_, A_Vspi_, and B).

We measured bite force in mice using a modified version of the device previously described by Okuda-Akabane et al. (1997). The appearance of the bite force measurement apparatus used in the present study was almost identical to the apparatus shown in Figures 1, 2 of the study (Okuda-Akabane et al., 1997). The bite force transducer consisted of two dental cobalt-chromium alloy palatal bars (length: 90 mm, width: 3.5 mm, and thickness: 1.3 mm), which served as pressure receptors when mice gnawed on the upper and lower incisors; two stainless steel cylindrical springs (outer diameter: 20 mm, width: 10 mm, and thickness: 1.5 mm, and a 3.6 mm wide slit); and four strain gauges (KFG-2-120-C1-11; Kyowa, Tokyo, Japan) that were affixed to the inner and outer surfaces of each cylindrical spring (Figures 1A,B). Two cylindrical springs were bonded to each end of the bite bar, such that the gap between the bite bars was 1.0 mm. When a vertical force is applied to the bite bars, the resistance of the strain gauge changes in proportion to the strength of the force. A full bridge was constructed with four gauges and connected to a strain amplifier (WGA-680A-00; Kyowa; Figure 1C). When the mouse bites the two palatal bars, the force is converted and amplified as a voltage value by the strain amplifier. Both ends of the upper bite bar were fixed to a post, such that the transducer was activated only when the mouse bit the bite bars with its upper and lower jaws; it was not activated when the mouse placed its mouth on the bar. The linearity of the load-to-output relationship of the biting force transducer was tested. The load-to-output voltage of the bite force transducer relationship was highly linear (*R*^2^ = 0.9996; Figure 1D).

We newly developed a closed-loop system in which the mice were provided water as a reward when they bit the bite force transducer with a force greater than the threshold (Figure 1E). The closed-loop system was developed using Bonsai software (Lopes et al., 2015; Buccino et al., 2018; Lopes and Monteiro, 2021). The EMG recording was amplified (EBA-100; Unique Medical) to optimal bandwidth (15–1,000 Hz). The outputs of the strain amplifier and EMG amplifier were digitized at 20,000 Hz using USB-6211 (National Instruments; Austin, TX, USA) and stored on a personal computer with Bonsai software. The bite force values were processed online in Bonsai; when the moving average for 1 s exceeded the threshold value of 30 g/s, the computer output a signal and activated the syringe pump to reward the mouse with water. The threshold value of 30 g/s was set as the upper limit, at which even mice with the weakest bite force could obtain the water reward.

Mice were subjected to EMG electrode implant surgery. After a recovery period of at least 1 week, mice were moved to a recording cage (clear acrylic cage with 20 cm height × 42 cm width × 25 cm depth,) connected to a recording cable, and then habituated for 30 min. The next day, after 24 h of drinking water restriction, the mice were again moved to a recording cage and connected to a recording cable. Then, bite force and EMG were recorded for 30 min. The mouse behavior was also recorded by two video cameras. During the 30-min recording interval, mice were allowed to move freely in the recording cage, chew the bite bar, drink the rewarded water, and eat the sunflower seeds in the recording cage without any restrictions.

### EMG and bite force analysis

The EMG activity of the masseter muscle was DC removed (time constant = 5 ms), rectified, and integrated (time constant = 10 ms) using Spike 2 software (version 8; Cambridge Electronic Design, Cambridge, UK). Mastication data for sunflower seeds were obtained from stable mastication cycles (i.e., five or more consecutive cycles). A double-threshold method (mean + 2 standard deviation (SD) of the quiet period and minimum duration of 10 ms (Bonato et al., 1998) was used to identify an EMG burst. Measured parameters were the mean duration, mean peak amplitude, mean interpeak interval, and mean muscle activity (i.e., area under the curve) of each masseter muscle burst (Ariyasinghe et al., 2004; Ootaki et al., 2004). The waveform of the bite was smoothed (time constant = 5 ms) using Spike 2 software. A double-threshold method (mean + 2 SD of the quiet period and minimum duration of 10 ms) was used to identify a bite. Measured parameters were the mean bite force (i.e., the peak amplitude of the curve) and the mean bite energy (i.e., area under the curve).

Further, we devised a new analysis, which we term correlative electromyography and force myography (CLEF) analysis, to estimate the bite force and energy during food chewing from the obtained bite force and EMG data. In the CLEF analysis, we first created an approximate equation from the scatter plots of muscle activity in EMG and bite force/energy during bar chewing. Using this approximate equation, we estimated bite force and energy applied to food from muscle activity in EMG during feeding.

### Hematoxylin and eosin staining and immunohistochemistry

Six 3 × Tg-AD and six NonTg mice (male, 3 months old) were deeply anesthetized using a mixture of medetomidine (0.3 mg/kg; Kobayashi Kako, Fukui, Japan), midazolam (4 mg/kg; Astellas Pharma, Tokyo, Japan), and butorphanol (5 mg/kg; Meiji Seika Pharma Co., Ltd., Tokyo, Japan). Then, they were transcardially perfused with 5 mL of 5 mM sodium phosphate-buffered 0.9% saline (PBS, pH 7.4), followed by 50 mL of 3% formaldehyde, 75% saturated picric acid, and 0.1 M Na_2_HPO_4_ (adjusted to pH 7.0 with NaOH). The brains and masseter muscles were removed and postfixed for 4 h at 23–25°C with the same fixative. The masseter muscles were sequentially dehydrated with increasing concentrations of 70–100% ethanol and replaced with xylene, and then embedded in paraffin. The paraffin-embedded masseter muscles were cut into 5-μm-thick sections using a microtome, and the sections were stained with hematoxylin and eosin.

The brains were cryoprotected with 30% sucrose in PBS, cut into 40-μm-thick coronal sections using a freezing microtome, and serially collected in PBS. Some sections were stained for Nissl bodies with cresyl violet to identify the cytoarchitectonic areas according to the mouse brain atlas of Paxinos and Franklin (2019). For immunoperoxidase staining, the brain sections obtained from 3 × Tg-AD and NonTg mice were incubated overnight with one of the following antibodies: mouse monoclonal anti-amyloid β (Aβ), 1–16 IgG_1_ (1:1,000 dilution; clone 6E10, SIG-39320, Covance, Princeton, NJ, USA), or rabbit monoclonal anti-phosphorylated (p) tau (S396) antibody (1:4,000 dilution; clone EPR2731, ab109390, Abcam, Cambridge, UK)—all in PBS containing 0.3% (v/v) Triton X-100, 0.12% (w/v) lambda-carrageenan, 0.02% (w/v) sodium azide, and 1% (v/v) normal donkey serum (PBS-XCD). After several washes in PBS containing 0.3% Triton X-100 (PBS-X), the sections were incubated for 2 h with 10 μg/mL biotinylated antibody to mouse IgG (AP192B; Merck Millipore) or rabbit IgG (BA-1000; Vector Laboratories, Burlingame, CA, USA) in PBS-XCD. After several washes in PBS-X, the sections were incubated for 1 h with avidin-biotinylated peroxidase complex (1:100; ABC-Elite, Vector Laboratories) in PBS-X. After rinses with PBS, the bound peroxidase was developed brown color after reaction for 30–60 min with 0.02% (w/v) 3,3′-diaminobenzidine-4HCl (DAB, 347-00904; Dojindo, Kumamoto, Japan) and 0.003% (v/v) H_2_O_2_ in 50 mM Tris-HCl, pH 7.6. Antibody specificities were determined using negative control sections that had been incubated without primary or secondary antibodies for each experiment. Immunoreactivity was not detected in the negative controls. All above incubations and reactions were performed at 23–25°C. The stained sections were thoroughly washed in PBS, mounted onto APS-coated slide glasses (APS-01; Matsunami Glass Ind, Osaka, Japan), air-dried, dehydrated in an ethanol series, cleared in xylene, and coverslipped for observation *via* microscopy.

For immunofluorescent staining, brain sections from 3 × Tg-AD and NonTg mice were incubated overnight with a mixture of affinity-purified goat polyclonal anti-choline acetyltransferase (ChAT) antibody (1:1,000 dilution; AB144P, Merck Millipore, Burlington, MA, USA) and affinity-purified rabbit polyclonal anti-vesicular glutamate transporter 1 (VGluT; 1 μg/mL; Hioki et al., 2003) in PBS-XCD. After several washes in PBS-X, the sections were incubated for 2 h with 5 μg/mL Alexa Fluor 488-conjugated antibody to goat IgG (ab150133; Abcam, Cambridge, MA, USA) and 5 μg/mL Alexa Fluor 594-conjugated antibody to rabbit IgG (ab150068; Abcam) in PBS-XCD. Antibody specificities were determined using negative control sections that had been incubated without primary or secondary antibodies for each experiment. Immunoreactivity was not detected in the negative controls. All above incubations and reactions were performed at 23–25°C. All the sections were washed thoroughly in PBS, mounted onto APS-coated slide glasses (APS-01; Matsunami Glass Ind), air dried, and coverslipped with 90% (v/v) glycerol and 2.5% (w/v) triethylendiamine (antifading agent) in 20 mM Tris-HCl, pH 7.4. For the analysis of VGluT1-immunopositive varicosities in the trigeminal motor nucleus (Vmo), z-stack images were captured under the confocal laser-scanning microscope (LSM 900; Zeiss, Oberkochen, Germany) with an oil-immersion 63 × objective lens (Plan Apochromat, numerical aperture = 1.4; Zeiss) and pinhole of 1.0 Airy unit.

### Statistical analysis

Sample sizes for behavioral, electrophysiological, and morphological analyses were deduced from previously published studies (Goto et al., 2020; Kim et al., 2021). We used the following mice to perform each experiment: for immunostaining of Aβ and p-tau, 2-month-old 3 × Tg-AD mice (*n* = 3), 3-month-old 3 × Tg-AD mice (*n* = 3), 2-month-old NonTg mice (*n* = 3), and 3-month-old NonTg mice (*n* = 3); for immunofluorescent staining of VGluT1 and ChAT, 3-month-old 3 × Tg-AD mice (*n* = 5) and 3-month-old NonTg mice (*n* = 4); and for bite force and EMG recording, 3–4-month-old 3 × Tg-AD mice (*n* = 10) and 3–4-month-old NonTg mice (*n* = 10) (mainly due to a broken wire, we could not record EMG from all animals). For histological analysis of the masseter muscles, we used 3-month-old 3 × Tg-AD mice (*n* = 7) and 3-month-old NonTg mice (*n* = 6). Bilateral masseter muscles were harvested from the mice used for the immunostaining of trigeminal nuclei and used in the experiments.

Experiments were conducted in a blinded manner with respect to genotype. For statistical analysis, such as two-way analysis of variance and *post-hoc* Tukey's multiple comparison test, and Student's *t*-test, GraphPad Prism 9 software (GraphPad Software, Inc., San Diego, CA, USA) and Excel (Microsoft, Redmond, WA, USA) were used. A *p*-value < 0.05 was considered statistically significant.

## Results

### Preferential AD pathology in the mesencephalic trigeminal nucleus of 3 × Tg-AD mice

The trigeminal nuclei (Figure 2A) constitute important brain regions for mastication. Thus, we performed immunohistochemistry for Aβ and p-tau on the trigeminal nuclei of 2- and 3-month-old 3 × Tg-AD male mice (*n* = 3 each) and 2- and 3-month-old NonTg male mice (*n* = 3 each). We observed the most prominent deposition of Aβ in the mesencephalic trigeminal nucleus (Vmes), and by 3 months of age, accumulation of intraneuronal Aβ was clearly observed not only in the Vmes but also in the Vmo (Figure 2B). In the principal sensory trigeminal nucleus (Vpri), there were a few scattered cell bodies with weak immunoreactivity for Aβ. Because the 6E10 antibody does not react with mouse Aβ, no immunoreactivity was observed in NonTg mice. In 2-month-old 3 × Tg-AD mice, only a few p-tau weakly positive cell bodies (Figure 2B; arrowheads) were observed in the Vmes. In 3-month-old 3 × Tg-AD mice, p-tau intensely positive Vmes-neuron axons and cell bodies (Figure 2B; arrows) were observed. In the Vmo, a number of p-tau-positive axon fibers were observed that might be derived from the Vmes. In other trigeminal nuclei, less p-tau immunoreactivity was observed. The antibody against p-tau used in the present study cross-reacts not only with human p-tau but also with mouse p-tau. However, no immunoreactivity was observed in 2- to 3-month-old NonTG mice, suggesting that endogenous tau was not hyperphosphorylated in 3-month-old NonTg mice. The results of the semi-quantitative analysis of p-tau immunoreactivity in the Vmes are shown in Supplementary Figure S1.

### Reduction of VGluT1-positive varicosities in the Vmo of 3 × Tg-AD mice

**Figure 3:**
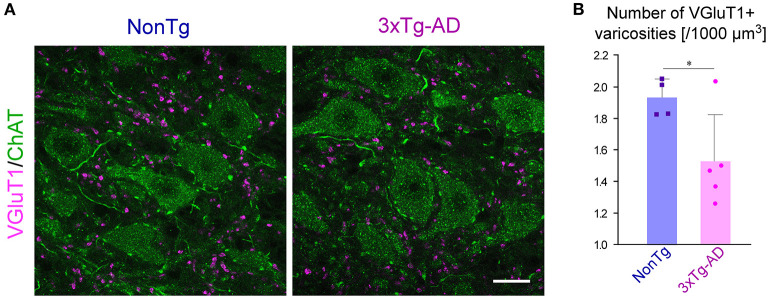
The density of VGluT1-immunopositive varicosities in the Vmo. **(A)** Representative confocal image of the center of dorsolateral Vmo. Motor neurons in the Vmo were labeled with ChAT immunoreactivity (green). In the Vmo, VGluT1-immunopositive varicosities (magenta) derived from the Vmes are located only in the dorsolateral part of the Vmo innervating the jaw-closing muscles. The density of VGluT1-immunopositive varicosities appeared to be decreased in 3 × Tg-AD mice than in NonTg mice. **(B)** The 3-month-old 3 × Tg-AD mice (*n* = 5) showed a significant reduction in the density of VGluT1-immunopositive varicosities compared to NonTg mice (*n* = 4) (*p = 0.0402, two-tailed unpaired *t*-test). Scale bar: 10 μm **(A)**.

We investigated whether the AD pathology observed predominantly in Vmes neurons affects the neural connections from the Vmes to the Vmo. Because almost all of the VGluT1-immunopositive axon terminals within the Vmo have been reported to originate from primary afferent neurons in the Vmes and to be distributed only in the dorsolateral part of the Vmo innervating jaw-closing muscles (Pang et al., 2009), we measured the density of VGluT1-immunopositive axon varicosities in the dorsolateral part of the Vmo. We performed double immunofluorescent staining for ChAT to visualize Vmo neurons and for VGluT1 (Figure 3A). Serial sections of the Vmo were prepared from 3-month-old 3 × Tg-AD mice (*n* = 5) and NonTg mice (*n* = 4); z-stack images were acquired on the section with the largest area of the dorsolateral Vmo. In the center of the dorsolateral Vmo, in a volume of 1,000,000 μm^3^ (25,000 μm^2^ area × 40 μm thickness), the number of VGluT1-positive axon varicosities was measured, and the density per unit volume was calculated. As shown in Figure 3B, the density of VGluT1-immunopositive axon varicosities in the dorsolateral Vmo was significantly reduced in 3 × Tg-AD mice compared to NonTg mice, suggesting that information input from Vmes neurons to Vmo neurons is reduced.

### Masticatory rhythms of 3 × Tg-AD mice are delayed compared with NonTg mice

**Figure 4:**
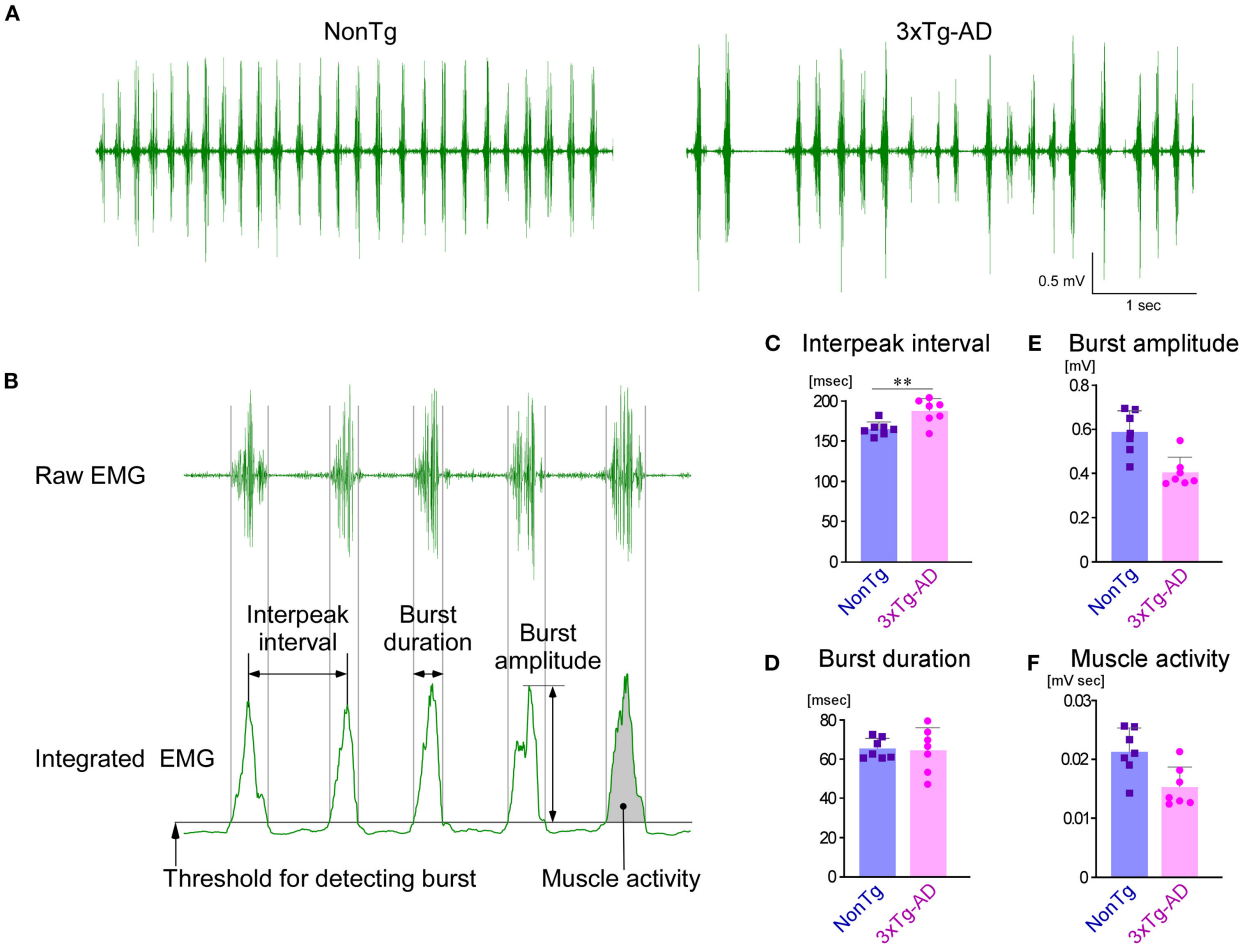
Comparison of EMG findings in the masseter muscles during sunflower seed chewing between NonTg and 3 × Tg-AD mice. **(A)** Representative raw EMG recordings of the masseter muscles in 3–4-month-old NonTg and 3 × Tg-AD mice. The rhythm of the chewing cycle was more irregular and slower in 3 × Tg-AD mice than in NonTg mice. **(B)** EMG findings in five or more consecutive chewing cycles were analyzed. The details of how each parameter was measured are provided in the Section Materials and methods. The 3 × Tg-AD mice (*n* = 7) showed a significantly longer mean interpeak interval of bursts than did NonTg mice (*n* = 7) (**C**; **P = 0.0065, two-tailed unpaired *t*-test), but there was no difference in the mean duration of individual bursts (**D**; *P* = 0.8775, two-tailed unpaired *t-*test). Because the amplitude of bursts **(E)** and muscle activity **(F)** were greatly affected by the state of the electrodes in the EMG, they could not be compared between groups.

Among the trigeminal nuclei, the Vmo and Vmes are particularly important for masticatory function. To investigate the effects of AD pathology observed in the Vmes and Vmo of 3-month-old 3 × Tg-AD mice on masticatory function, we recorded and compared EMG of the masseter muscles and bite force in 3 × Tg-AD and NonTg mice aged 3–4 months. First, we analyzed EMG findings in the masseter muscle, recorded while mice were freely feeding on sunflower seeds (Figure 4A). Because the mastication cycle of 3 × Tg-AD mice appeared to be irregular and slower than the mastication cycle of NonTg mice in the EMG during feeding, we performed a detailed comparative analysis of EMG findings. Analysis was conducted focusing on the masticatory movements that were repeated more than five times consecutively (Figure 4B). The mean interpeak interval (Figure 4C) was significantly longer in 3 × Tg-AD mice than in NonTg mice, whereas the mean burst duration (Figure 4D) was not different, suggesting that the mastication cycle was delayed in 3 × Tg-AD mice. The mean burst amplitude (Figure 4E) and mean muscle activity (Figure 4F) were lower in 3 × Tg-AD mice than in NonTg mice. Burst amplitude and muscle activity in EMG are positively correlated with muscle force (Roberts and Gabaldón, 2008; Disselhorst-Klug et al., 2009; Zhu et al., 2017); however, they are greatly affected by electrode conditions. Therefore, we could not assume that 3 × Tg-AD mice had a lower bite force than NonTg mice solely because the mean burst amplitude and mean muscle activity of the EMG findings in 3 × Tg-AD mice were lower than those of NonTg mice. Thus, we measured the actual occlusal forces of these mice.

### Bite force is lower in 3 × Tg-AD mice than in NonTg mice

To compare the bite force between 3 × Tg-AD and NonTg mice, we developed a closed-loop system in which mice were provided water as a reward when they chewed the bite force transducer; we then measured their voluntary bites. Mice were subjected to drinking water restrictions for 24 h from the previous day, and then transferred to recording cages. In the recording cage, a cable for recording EMG was connected to a connector placed on the head of each mouse. Mice could freely explore the recording cage, chew bite bars, drink reward water, and eat sunflower seeds in the recording cage without any restrictions. The recording was conducted for 30 min.

**Figure 5:**
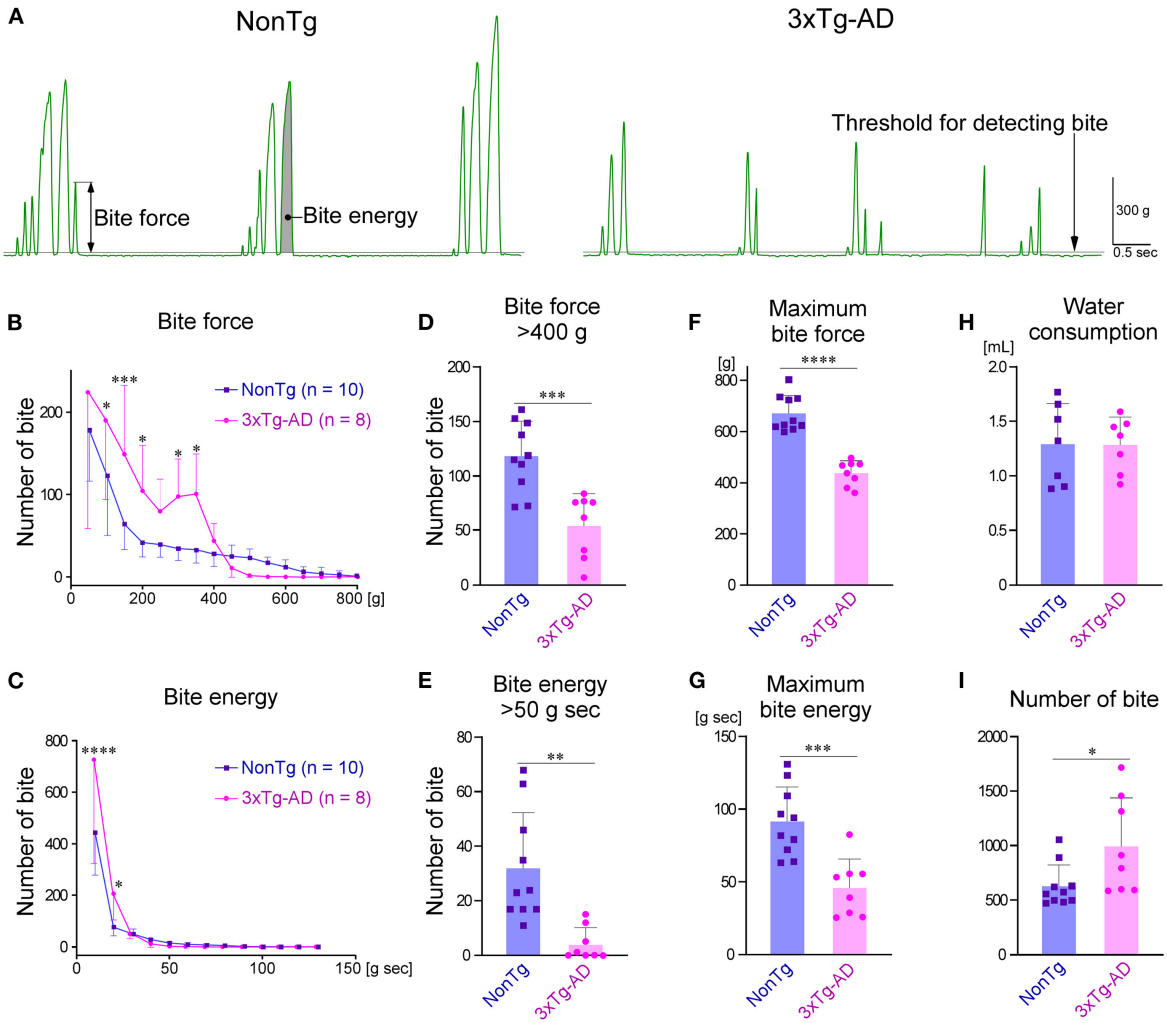
Comparison of bite force between 3–4-month-old NonTg and 3 × Tg-AD mice when chewing bite bars. **(A)** Representative raw bite force recordings of a NonTg mouse and a 3 × Tg-AD mouse. The details of how each parameter was measured are provided in the Section Materials and methods. The distribution of bite force during the 30-min measurement was significantly different between 3 × Tg-AD and NonTg mice at bite forces of 100–200 g and 300–350 g (**B**; *P* = 0.0192 at 100 g, *p* = 0.0008 at 150 g, *p* = 0.0396 at 200 g, *p* = 0.0353 at 300 g, and *p* = 0.0163 at 350 g by Bonferroni multiple comparison tests). In addition, the distribution of bite energy during the 30-min measurement was significantly different at bite energies of 10–20 g sec (**C**; *p* < 0.0001 at 10 g sec, and *p* = 0.0194 at 20 g sec by Bonferroni multiple comparison tests). Compared with 3 × Tg-AD mice, NonTg mice had a significantly higher frequency of occlusions with a bite force of more than 400 g (**D**; ***p = 0.0005, two-tailed unpaired *t*-test) and bite energy of more than 50 g sec (**E**; **p = 0.0018, two-tailed unpaired *t*-test). Furthermore, the maximum bite force (**F**; ****p < 0.0001, two-tailed unpaired *t*-test) and maximum bite energy (**G**; ***p = 0.0005, two-tailed unpaired *t*-test) were significantly greater in NonTg mice than in 3 × Tg-AD mice. Although there was no difference in the amount of water consumed during the 30-min measurement between 3 × Tg-AD and NonTg mice (**H**; *p* = 0.9737, two-tailed unpaired *t*-test), the number of bites was significantly higher in 3 × Tg-AD mice (**I**; *p = 0.0307, two-tailed unpaired *t*-test).

The raw waveform of the bite force measured by the bite force transducer is shown in Figure 5A. The peak value of the waveform was defined as the bite force of each occlusion. The area under the curve was defined as the bite energy of each occlusion. The distributions of bite force and energy during the 30-min recording are shown in Figures 5B,C, respectively. The number of bites with small force or small energy was significantly greater in 3 × Tg-AD mice than in NonTg mice. Comparing the frequency of biting with a force of more than 400 g or energy of more than 50 g sec between 3 × Tg-AD and NonTg mice, we found that the bite frequency was significantly lower in 3 × Tg-AD mice in both cases (Figures 5D,E). During the 30-min measurement, the maximum bite force and maximum bite energy when chewing the bars of the bite force transducer were significantly smaller in 3 × Tg-AD mice than in NonTg mice (Figures 5F,G). These results indicated that the bite force was lower in 3 × Tg-AD mice than in NonTg mice. During the 30-min recording, there was no difference in the amount of water consumption by either group (Figure 5H), but the number of bites (Figure 5I) was significantly higher in 3 × Tg-AD mice than in NonTg mice. These results suggest that 3 × Tg-AD mice compensate for their weak bite force by chewing more often than do NonTg mice.

### CLEF analysis of bite force during sunflower seed feeding

**Figure 6:**
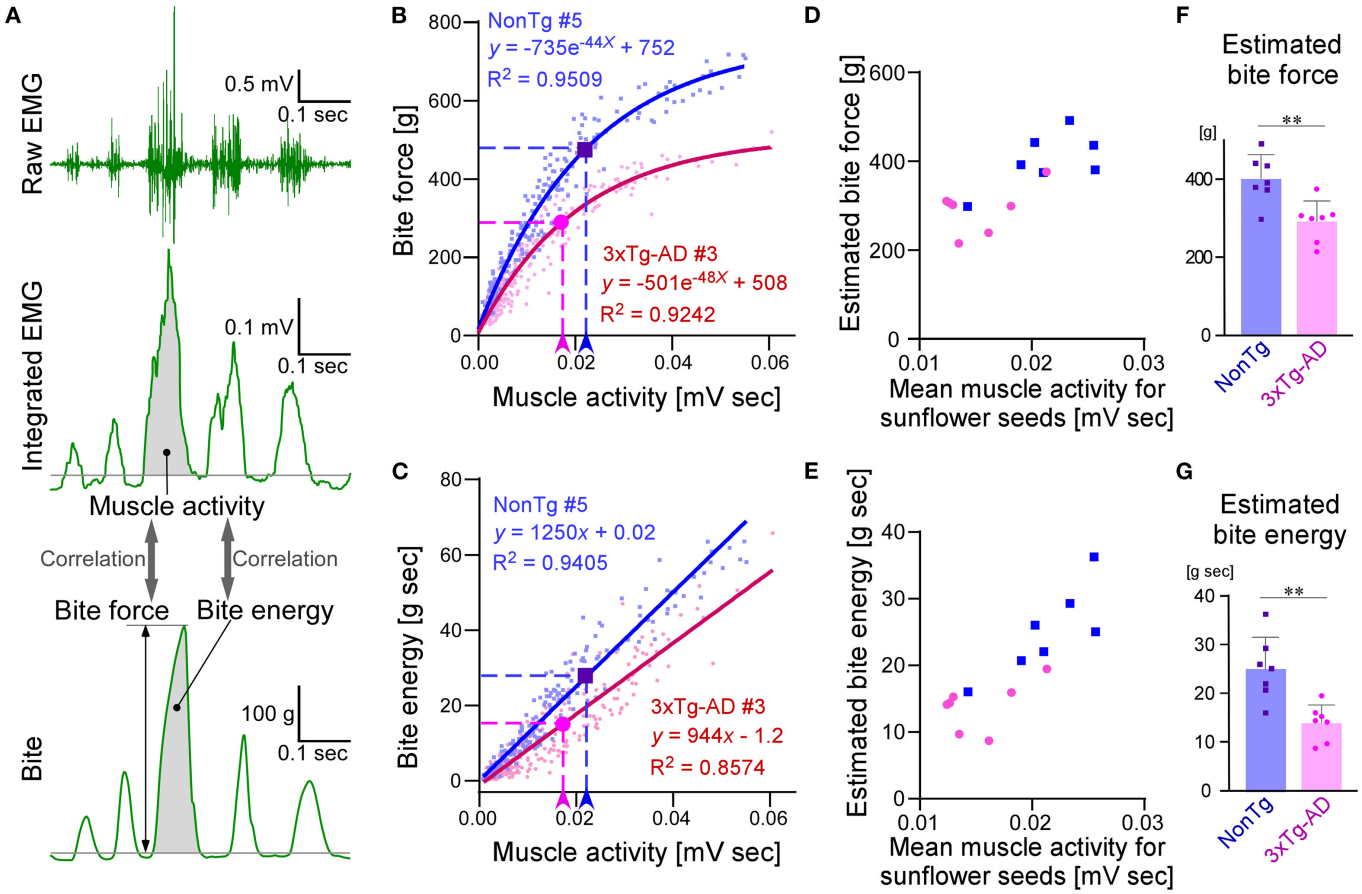
Estimation and comparison of bite force and bite energy during sunflower seed feeding between 3–4-month-old NonTg and 3 × Tg-AD mice by CLEF analysis. Representative waveforms of raw EMG findings in the masseter muscle, integrated EMG, and bite when mice chewed bite bars **(A)**. CLEF analysis between muscle activity and bite force or energy was performed on 14 mice, seven each of NonTg and 3 × Tg-AD mice. Scatter plot and nonlinear regression curves showing the correlation between muscle activity and bite force when NonTg mouse #5 (**B**; blue dots and solid curve) and 3 × Tg-AD mouse #3 (**B**; red dots and solid curve) chewed bite bars. Arrowheads and dotted lines indicate the mean value of muscle activity when eating sunflower seeds and the biting force estimated from it, respectively **(B)**. Scatter plot and linear regression lines showing the correlation between muscle activity and bite energy when NonTg mouse #5 (**C**; blue dots and solid curve) and 3 × Tg-AD mouse #3 (**C**; red dots and solid curve) chewed bite bars. Arrowheads and dotted lines indicate the mean value of muscle activity when eating sunflower seeds and the biting energy estimated from it, respectively **(C)**. Scatter plots and nonlinear regression curves or linear regression lines for the other 12 mice, not shown here, are provided in Supplementary Figure S2. Scatter plots of mean muscle activity while chewing sunflower seeds and estimated bite force **(D)** or energy **(E)** in NonTg (blue dots) and 3 × Tg-AD (red dots) mice, seven mice each. 3 × Tg-AD mice had significantly lower estimated bite force (**F**; **p = 0.0038, two-tailed unpaired *t*-test) and energy (**G**; **p = 0.0020, two-tailed unpaired *t*-test) during sunflower seed chewing, compared with NonTg mice.

Next, we investigated whether any differences in the bite force and energy were present in a more natural situation, for example, when mice were eating sunflower seeds. It is difficult to directly measure the occlusal force applied to food during consumption. Therefore, we conducted CLEF analysis of the simultaneously recorded data of EMG and bite force. The representative waveforms of bite force and EMG, measured simultaneously while the mice chewed bite bars, are shown in Figure 6A. When bite force and energy were plotted against EMG muscle activity, the two parameters showed a strong correlation, and they were approximated by exponential and linear equations, respectively. Figures 6B,C show the representative plots of bite force and energy against EMG muscle activity, respectively. We analyzed the correlations between EMG muscle activity and bite force or bite energy in seven 3 × Tg-AD mice and seven NonTg mice, and we found that they all showed strong correlations. For each mouse, we drew an approximation curve and obtained the approximate equation, which was unique for each mouse (Figures 6B,C; Supplementary Figure S2). Using these unique approximations, we estimated the bite force and energy of chewing sunflower seeds from the mean muscle activity of each mouse while it chewed the sunflower seeds (Figures 6D,E). The results showed that 3 × Tg-AD mice had significantly lower estimated bite force and energy when chewing sunflower seeds than NonTg mice (Figures 6F,G). In summary, 3 × Tg-AD mice had a delayed chewing rhythm and lower bite force, compared with NonTg mice.

### The masseter muscle has no morphological changes in 3 × Tg-AD mice

**Figure 7:**
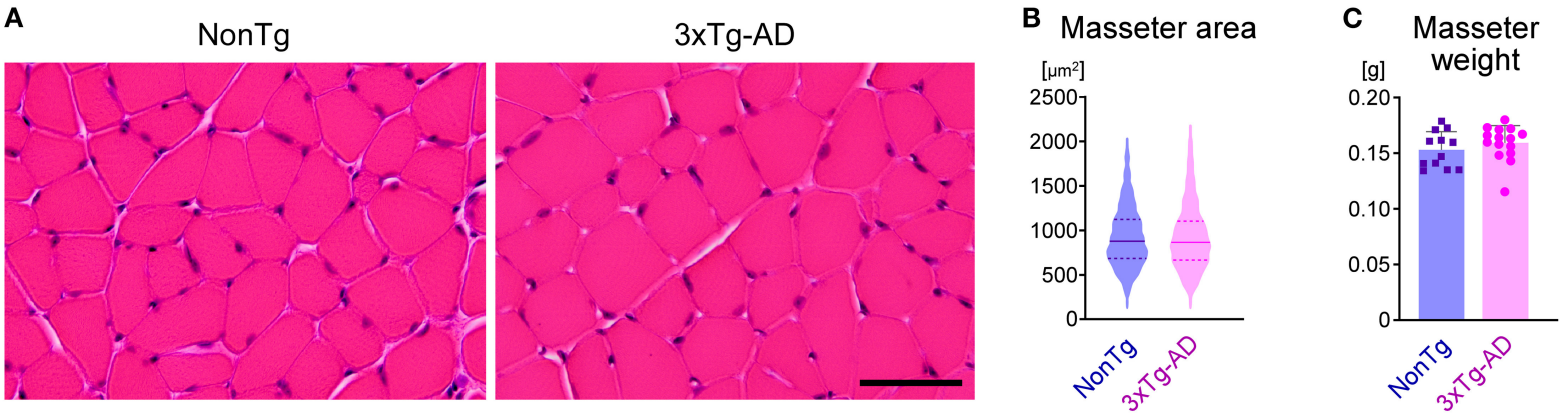
Morphological comparison of the masseter muscle between 3-month-old NonTg and 3 × Tg-AD mice. **(A)** Hematoxylin and eosin staining of transverse sections of the masseter muscles in 3-month-old NonTg and 3 × Tg-AD mice. **(B)** Violin plot depicting the cross-sectional area of muscle fibers of the masseter muscle. The cross-sectional area of 100 muscle fibers from each of six NonTg mice (total 600 muscle fibers) and 100 muscle fibers from each of six 3 × Tg-AD mice (total 600 muscle fibers) was randomly selected and measured. The horizontal solid line and the pair of horizontal dotted lines indicate the mean and the interquartile range, respectively. There were no significant differences between 3-month-old 3 × Tg-AD and NonTg mice in the mean cross-sectional area of muscle fibers of the masseter muscle (**B**; *p* = 0.9333, two-tailed unpaired *t*-test) or the mean weight of the masseter muscle (**C**; *p* = 0.3582, two-tailed unpaired *t*-test; *n* = 12, NonTg; *n* = 14, 3 × Tg-AD). Scale bar: 50 μm **(A)**.

Decreased bite force may be caused by muscle abnormalities (e.g., muscle atrophy and sarcopenia) (Carter et al., 2010), or lesions in neurons that control the masticatory muscles, or abnormalities in both muscle and neurons. To check for abnormalities in the masseter muscle, we compared the mean cross-sectional area of muscle fibers (Figures 7A,B) and the mean weight of the masseter muscle (Figure 7C) between 3-month-old 3 × Tg-AD (*n* = 7) and NonTg mice (*n* = 6). There was no significant difference in masseter muscle morphology between 3 × Tg-AD and NonTg mice. Furthermore, the body weights of 3-month-old 3 × Tg-AD and NonTg mice were 24.6 ± 1.5 g (mean ± SD) and 25.0 ± 1.5 g, respectively (*p* = 0.6108; *n* = 8 each; two-tailed unpaired *t*-test), indicating that there were no differences in nutritional status between groups. These results suggest that the decreased biting force and delayed mastication rhythm observed in 3 × Tg-AD mice were not caused by abnormalities in the masseter muscle.

## Discussion

In the present study, immunohistochemistry of the trigeminal nuclei revealed that the most intense Aβ- and p-tau immunoreactivities were present in the Vmes among the trigeminal nuclei of 3 × Tg-AD mice at 3 months of age. In addition, reduced axonal projections from the Vmes to the Vmo were suggested. Since the Vmes plays an important role in the regulation of masticatory function, neurological damage in the Vmes can affect mastication. In order to quantitatively analyze bite force, one of the evaluation items for oral frailty, in mice, we developed a system to simultaneously record bite force and EMG of the masseter muscle and proposed a new analysis named CLEF analysis to estimate bite force and energy during food chewing in mice. The bite force and energy of 3 × Tg-AD mice while eating sunflower seeds were smaller than the bite force and energy of NonTg mice. Analysis of EMG also showed that the mastication rhythm was delayed in 3 × Tg-AD mice. However, there was no difference in the weight of masseter muscles or the cross-sectional area of muscle fibers between 3 × Tg-AD and NonTg mice, suggesting that the decreased bite force and delayed mastication rhythm observed in 3 × Tg-AD mice were not due to abnormalities in the masseter muscle.

In the present study, we developed a closed-loop system in which the mice were provided water as a reward when they bit the bite force transducer with a force greater than the threshold (Figure 1). Using this system, we measured the bite force when chewing bite bars, we found a significant difference between 3 × Tg-AD and NonTg mice. However, individual mice may have distinct motivational or emotional strengths because of differences in thirst, which may affect bite force (Kuchiiwa and Kuchiiwa, 2014). Therefore, we conducted a new analysis, CLEF, which can quantitatively evaluate bite force and energy during natural food intake. However, CLEF has a technical limitation in that the bite force transducer measures occlusal force *via* the incisors. Although we revealed an approximation of the correlation between the bite force or energy of the incisors and the muscle activity of EMG, the approximation may be different from that of the molars. When chewing sunflower seeds continuously in a rhythm, mice would use their molars. Thus, the estimated bite force and energy during sunflower seed chewing may be slightly different from the actual values. However, the fact that 3 × Tg-AD mice at 3–4 months of age have reduced bite force during food mastication (Figure 6) is unlikely to be overturned. Furthermore, the decrease in maximum bite force and energy when chewing bite bars (Figure 5) and the delay in mastication rhythm in EMG during food chewing (Figure 4) indicate deterioration of masticatory function in 3–4-month-old 3 × Tg-AD mice, compared with NonTg mice. In summary, our method allows the estimation of bite force and energy in the natural state of laboratory animals, along with quantitative comparison between individuals.

By combining quantitative bite analysis with histochemical analysis, which is highly invasive and cannot be performed in humans, we were able to comprehensively analyze the pathological mechanism of oral frailty in mice. We found that AD pathology was particularly intense in the Vmes, which is important for controlling masticatory movement (Dessem and Taylor, 1989; Hunter et al., 2001; Lazarov, 2007) in 3-month-old 3 × Tg-AD mice (Figure 2). The result is consistent with previous findings regarding AD pathology in the brainstem of 3 × Tg-AD mice. Accumulation of intraneuronal Aβ has been reported to be observed in the brainstem of 3 × Tg-AD mice at 2 months of age (Overk et al., 2009; Goto et al., 2020), prior to the hippocampus and amygdala. In 3 × Tg-AD mice older than 4 months of age, AD pathology appears in brain regions other than the brainstem, such as the hippocampus, amygdala, and cerebral cortex (Oddo et al., 2003a,b; Billings et al., 2005), and this would affect mastication. Therefore, in the present study, mice older than 4 months of age were not included in the analysis of mastication in order to exclude the effects of AD pathology in the hippocampus, amygdala, and cerebral cortex.

The pathology of AD, such as intraneuronal accumulation of Aβ and excessive tau phosphorylation, causes neuronal dysfunction. For example, intraneuronal accumulation of Aβ in the cerebral cortex and hippocampus of 3 × Tg-AD mice is observed after 4 months of age, leading to spatial memory impairment (Oddo et al., 2003a,b, 2006; Billings et al., 2005; Caccamo et al., 2006). Aβ oligomers trigger synaptic degeneration (Jang et al., 2021). Overexpression and hyperphosphorylation of tau impair the localization and distribution of mitochondria (Ebneth et al., 1998; Kopeikina et al., 2011; Shahpasand et al., 2012; Rodríguez-Martín et al., 2013), leading to defects in axonal function and loss in synapses (Cabezas-Opazo et al., 2015; Wang et al., 2015). AD pathology causes a decrease in synaptic density, which is strongly correlated with neurological dysfunctions (Terry et al., 1991; Scheff and Price, 2003; Calignon et al., 2012; Jang et al., 2021). Indeed, the density of VGluT1-positive axon terminals derived from the Vmes was reduced in the Vmo of 3-month-old 3 × Tg-AD mice (Figure 3).

In contrast to the AD pathology observed in the Vmes and Vmo of 3 × Tg-AD mice and the resulting changes in axon terminals, no abnormalities were observed in the masseter muscle. We observed no significant differences in the cross-sectional area of muscle fibers and masseter muscle weight between 3-month-old 3 × Tg-AD and NonTg mice (Figure 7). This result is consistent with previous studies in which no findings suggestive of sarcopenia have been observed in young (2–4 months old) 3 × Tg-AD mice (Monteiro-Cardoso et al., 2015; Xu et al., 2022). Therefore, the decreased oral function observed in 3 × Tg-AD mice at 3–4 months of age is not due to muscle abnormalities, but rather to the AD pathology of the Vmes and Vmo. In the present study, we evaluated AD pathology and masticatory function only in 3- to 4-month-old mice. In the future, the relationship between AD pathology and the degree of decline in masticatory function will be analyzed over time, and similar analyses will be conducted in other AD model mice to elucidate the mechanism of oral weakness in more detail.

Recent clinical studies have shown that there is a close relationship between cognitive function and oral frailty, but the causal relationship remains unclear. Cohort studies have shown that oral deterioration (e.g., periodontal disease and edentulous jaws) is a risk factor for dementia (Campos et al., 2017; Ikebe et al., 2018; Kim et al., 2020; Dibello et al., 2021a,b; Nakamura et al., 2021a,b). Reduced stimulation from the teeth to the brain because of oral deterioration and periodontal disease-related chronic inflammation may exacerbate AD. Conversely, the decline in cognitive function because of AD progression may contribute to oral deterioration. The results of the present study and the recent study by Kim et al. (2021) suggest that the Vmes and Vmo are damaged by AD pathology, thereby directly impairing masticatory function. Using CLEF analysis developed in the present study, we were able to quantitatively evaluate the decline in bite force and energy, a component of oral frailty, in mice and to analyze its relationship with AD pathology. CLEF analysis can also be useful in studies investigating the impact of reduced masticatory function on AD pathology. Thus, CLEF is a useful tool for clarifying the causal relationship between oral frailty and cognitive dysfunction.

## Conclusion

The present study clearly demonstrates the impact of AD pathology in the Vmes and Vmo on masticatory function by quantitative evaluation of food mastication using a novel CLEF analysis. Intracellular accumulation of Aβ and excessive phosphorylated tau were observed in the Vmes of 3-month-old 3 × Tg-AD mice. In addition, a decrease in the number of axons projecting from the Vmes to the Vmo was found, while no histological changes were observed in the masseter muscle. Therefore, it is concluded that the decreased masticatory function observed in 3–4-month-old 3 × Tg-AD mice is due to AD pathology in Vmes and Vmo. Our novel quantitative analyses of masticatory function using a mouse model of AD enabled a comprehensive understanding of oral frailty pathogenesis.

## Data availability statement

The datasets presented in this study can be found in online repositories. The names of the repository/repositories and accession number(s) can be found in the article/Supplementary material.

## Ethics statement

The animal study was reviewed and approved by the Committees for Animal Care and the Recombinant DNA Study at Kagoshima University.

## Author contributions

EK: experimental design, development of a closed-loop system, funding, animal harvesting, the performance of animal experiments, image quantification, figure creation, statistical analysis, and writing and editing of the manuscript. AK: development of a closed-loop system, animal harvesting assistance, immunohistochemistry, CLEF analysis, and editing of the manuscript. TY: CLEF analysis assistance and editing of the manuscript. HK: development of closed-loop system and editing of the manuscript. S-EM, HH, YO, HI, and AY: animal harvesting assistance and editing of the manuscript. TG: funding, figure creation assistance, statistical analysis, and writing and editing of the manuscript. All authors contributed to the article and approved the submitted version.

## Funding

This study was supported by Grants-in-Aid from the Ministry of Education, Culture, Sports, Science and Technology (MEXT) for Scientific Research (JP19K10058, JP22K09916 to EK, JP20K10296 to TG), for Promotion of Joint International Research [Fostering Joint International Research (A)] (JP19KK0419 to EK), and for Scientific Research on Innovative Areas (JP17H06311, JP22H05162 to EK).

## Conflict of interest

The authors declare that the research was conducted in the absence of any commercial or financial relationships that could be construed as a potential conflict of interest.

## Publisher's note

All claims expressed in this article are solely those of the authors and do not necessarily represent those of their affiliated organizations, or those of the publisher, the editors and the reviewers. Any product that may be evaluated in this article, or claim that may be made by its manufacturer, is not guaranteed or endorsed by the publisher.
