# Supplementary figures and images for: Development of a system to analyze oral frailty associated with Alzheimer's disease using a mouse model
Source: Front Aging Neurosci. 2022 Aug 2;14:935033. doi: 10.3389/fnagi.2022.935033 (PMC9380890; doi:10.3389/fnagi.2022.935033)

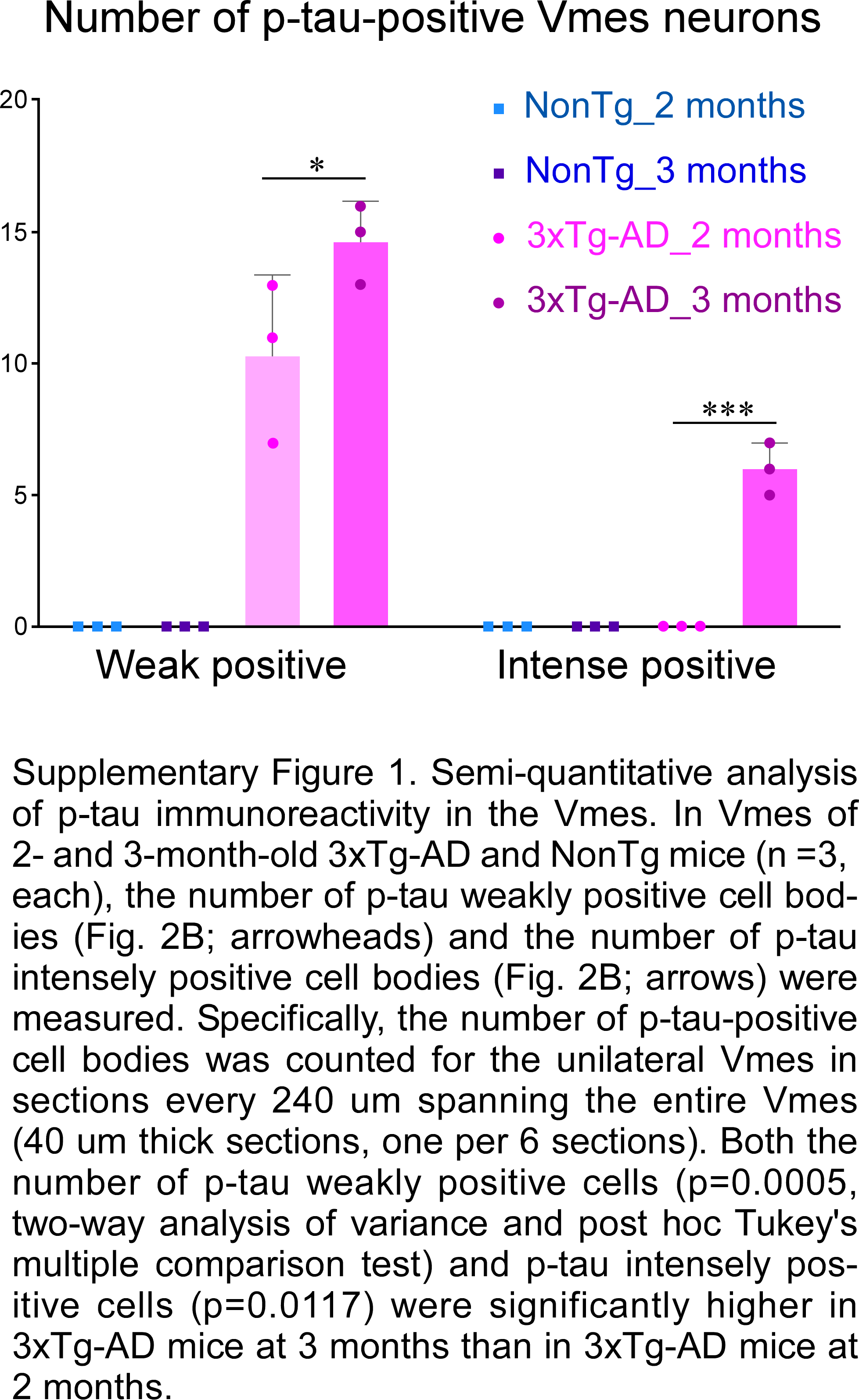

Supplement: Supplementary file 1 [file Image_1.TIF]

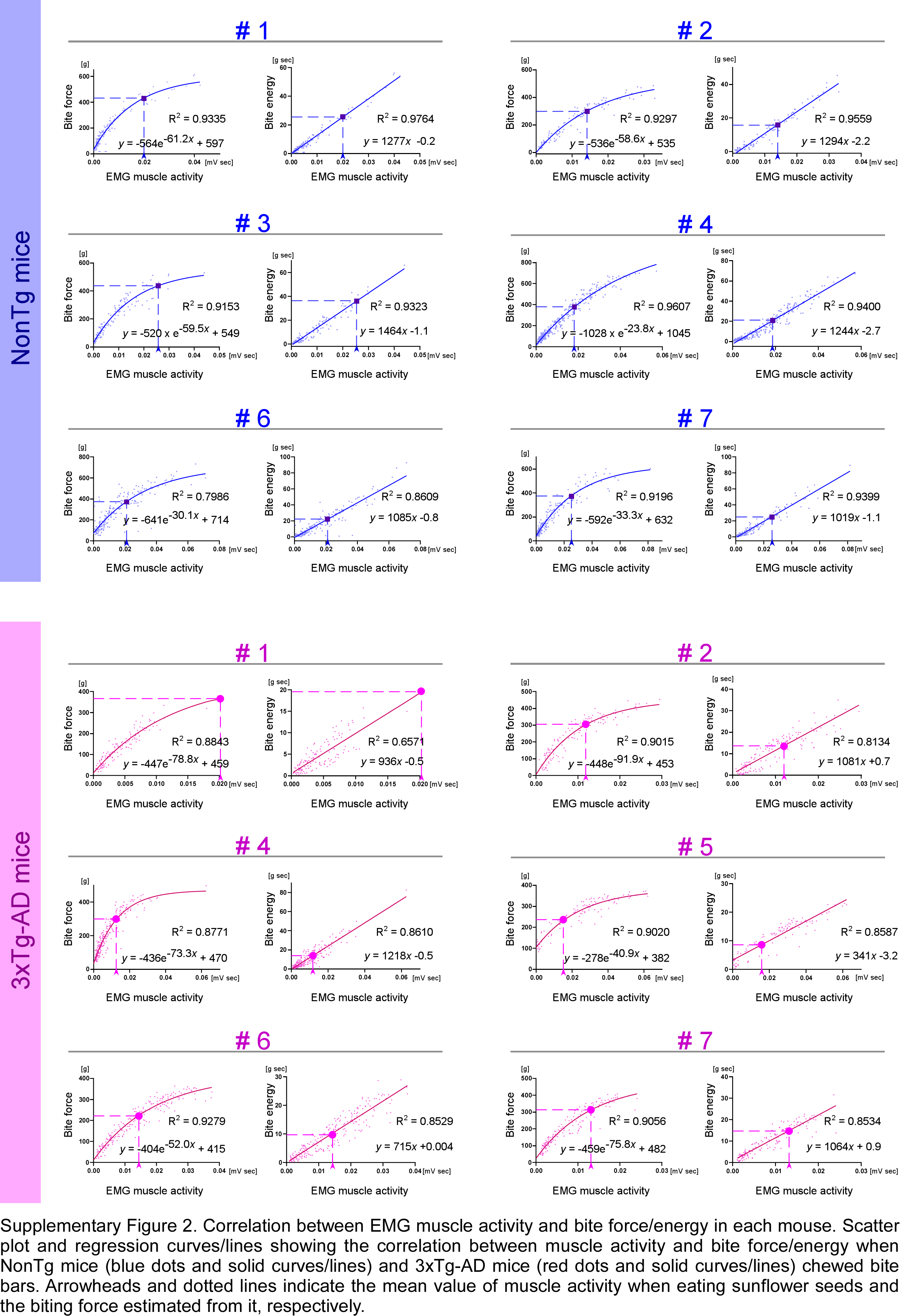

Supplement: Supplementary file 2 [file Image_2.TIF]
